# Darier-Roussy Subcutaneous Sarcoidosis Masquerading as Multiple Abscesses: A Report of a Rare Case

**DOI:** 10.7759/cureus.61959

**Published:** 2024-06-08

**Authors:** Yusuf A Ahmed, Walaa H Yusuf, Abdulla J Almubarak, Manar A Ali, Samar Tharwat

**Affiliations:** 1 Faculty of Medicine, Mansoura University, Mansoura, EGY; 2 Rheumatology and Immunology Unit, Department of Internal Medicine, Faculty of Medicine, Mansoura University, Mansoura, EGY

**Keywords:** case report, sarcoidosis, multiple abscesses, darier–roussy, subcutaneous sarcoidosis

## Abstract

Sarcoidosis is an idiopathic multisystemic granulomatous disease that mainly affects the lungs. Darier-Roussy subcutaneous sarcoidosis is among the specific and least encountered skin manifestations of sarcoidosis. In this case study, we report how subcutaneous sarcoidosis could mimic multiple abscesses presentation and hinder reaching a definitive diagnosis. A 65-year-old female presented with five, multiple, deep-seated skin lesions on the forearm, chest, and scalp. The lesions showed redness and tenderness. The patient also experienced arthralgia in the right ankle. Laboratory workup of the patient showed a high erythrocyte sedimentation rate (ESR), C-reactive protein (CRP), and white blood cell (WBC) count. The patient was suspected to have multiple abscesses, which were managed with antibiotics with no response. Thus, a computed tomography (CT) scan of the chest was done and showed mediastinal lymphadenopathy. A biopsy was taken from one of the right forearm skin lesions, and it revealed characteristic features consistent with sarcoidosis. The patient was managed with hydroxychloroquine and a tapering dose of prednisone. Therefore, subcutaneous sarcoidosis should be included in the differential diagnosis of subcutaneous lumps.

## Introduction

Sarcoidosis is an idiopathic multisystemic granulomatous disease. It was discovered by Besnier et al. in 1889 [1]. Sarcoidosis affects people of all ethnicities at any time during life, mostly females of North American, Indian, and European origin between the ages of 30 to 50 years. The underlying mechanisms of the disease can be linked to environmental factors and genetic predispositions [2]. The worldwide incidence of sarcoidosis is between 2.3 and 11 per 100,000, and its prevalence varies from 2.17 to 160 per 100,000 individuals [1].

Sarcoidosis can follow two different courses: a time-limited course or a chronic course. Lymphatics and lungs are the most frequently affected sites by sarcoidosis and usually present with hilar lymphadenopathy, pulmonary reticular pattern, and involvement of extrapulmonary organs, including the eyes, skin, and joints [3,4]. Skin is the second or third most affected organ [1], skin manifestations can present as papules, plaques, nodules, lupus pernio, scars, and ulceration, which can occur at any stage of sarcoidosis. Erythema nodosum is the most common cutaneous presentation presenting classically as tender subcutaneous nodules over the anterior tibia [3].

Subcutaneous sarcoidosis or Darier-Roussy is a rare finding among patients presenting with skin involvement. Recent studies suggest the incidence rate of subcutaneous sarcoidosis in patients with skin manifestations to be between 11.8% and 16% [5]. Subcutaneous sarcoidosis can present clinically as non-tender fleshy nodules, which range in size between 0.5 and 2 cm. Histologically, the nodules appear as non-caseating granulomas within the subcutaneous tissue. The ultrasonographic appearance of subcutaneous sarcoidosis is in the form of an irregularly defined mass with mixed hyperechoic and hypoechoic areas [5]. In this case study, we report how Darier-Roussy can have similar manifestations as multiple abscesses by clinical examination, imaging, and laboratory workup.

## Case presentation

**Figure 1:**
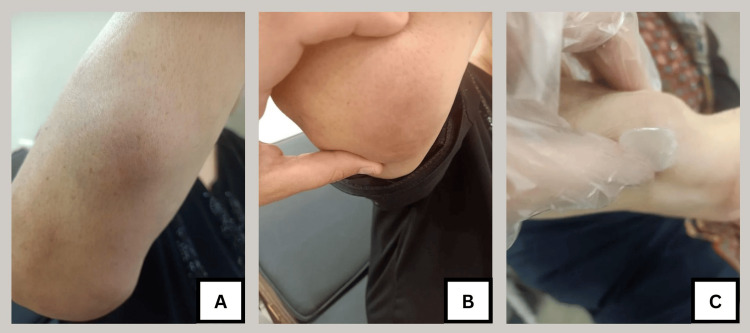
Multiple subcutaneous nodules of the right forearm A: Subcutaneous nodule measuring 2x3 cm at the upper part of the right forearm; B: Subcutaneous nodule measuring 5x6 cm at the extensor surface of the right elbow; C: Subcutaneous nodule measuring 4x5 cm at the lower part of the right forearm

A 65-year-old female presented with five, multiple, deep-seated skin nodules that were gradually increasing in size for a period of one month. On examination, three out of the five nodules were located on the proximal and distal parts of the right forearm (Figure 1), the other two nodules were found on the chest and scalp. The nodules showed slight redness and tenderness. The patient also experienced arthralgia in the right ankle joint. The patient did not have lymphadenopathy, chest or systemic manifestation, and did not have diabetes or hypertension. The laboratory workup of the patient showed a high erythrocyte sedimentation rate (ESR), C-reactive protein (CRP), and white blood cell (WBC) count with no lymphopenia (Table 1).

**Table 1:** Laboratory parameters of the subcutaneous sarcoidosis patient during diagnostic workup

Parameter	Result	Reference Range
Erythrocyte sedimentation rate	130 mm/h	< 30 mm/h
C-reactive protein	96 mg/dL	< 0.3 mg/dL
White blood cell count	11,500 cells/µL	4,000-11,000 cells/µL
Lymphocyte count	1,510 cells/µL	1,000-5,000 cells/µL
Angiotensin-converting enzyme	92 IU/L	13.3-63.9 IU/L

Ultrasound was needed to evaluate the nodules and the lesion was found to be hypoechoic. Thus, based on the findings of the examination alongside the laboratory workup and ultrasound findings the patient was suspected to have multiple abscesses. The patient was prescribed oral amoxicillin/clavulanic acid 1 gm every 12 hours for 10 days by a general practitioner. Then, she sought the care of a dermatology clinic and was prescribed levofloxacin 750 mg once per day for another 10 days. However, this did not result in any improvement in the nodules.

The patient was referred to the rheumatology department for further investigations to reach a definitive diagnosis. The autoimmune profile of the patient showed negative antinuclear antibody (ANA), anti-double-stranded deoxyribonucleic acid antibodies (anti-dsDNA), and low anti-streptolysin O titer. Also, angiotensin-converting enzyme (ACE) was ordered and found raised at 92 U/L, which is considered a marker of sarcoidosis (Table 1).

A computed tomography (CT) scan of the chest was done, which showed mediastinal lymphadenopathy that raised suspicion for sarcoidosis. Thus, to confirm the diagnosis, a biopsy was taken from one of the right forearm skin nodules; the nodule was firm and it did not contain any pus to be aspirated by fine-needle aspiration cut. Therefore, a biopsy of the lesion was sent for histopathology, and it revealed deep dermal and subcutaneous nodules of epithelioid histocytes admixed with multinucleated giant cells with surrounding few lymphocytes. The dermis and epidermis showed no remarkable changes. The result of non‐caseating epithelioid granulomas is consistent with subcutaneous sarcoidosis. Interferon-gamma release assay was used to exclude tuberculosis, as it is one of the main differentials of granulomatous diseases.

Hence, the patient was managed with hydroxychloroquine 400 mg/day for three months and a tapering dose of prednisone 1mg/kg/day (60 mg) once daily for three months. After two weeks of treatment, the subcutaneous nodules and arthralgia showed improvement. Moreover, ESR and CRP were normalized.

## Discussion

Cutaneous sarcoidosis can be categorized into specific and nonspecific forms. The nonspecific forms include erythema nodusum, calcinosis cutis, digital clubbing, and prurigo. On the other hand, macules/papules, plaques, lupus pernio, scar sarcoidosis, and subcutaneous nodules are considered specific skin findings with subcutaneous sarcoidosis being the least common [6]. Subcutaneous sarcoidosis is most frequently found in females, with the most common site of affection being the upper and lower limbs. The presence of abnormal chest radiographs is highly associated with subcutaneous sarcoidosis as the presence of lymphadenopathy and/or lung infiltrates was noted in 77.2% of the affected patients [5]. Other extra-cutaneous sarcoidosis manifestations associated with subcutaneous nodules include arthritis, mucositis, uveitis, dactylitis, parotitis, peripheral neuropathy, and cardiac and renal sarcoidosis [7,8]. In the present case, two extracutaneous manifestations were noted, which included mediastinal lymphadenopathy and arthralgia. ACE levels were found to be elevated in 68.3% of patients having subcutaneous sarcoidosis [5], which is also noted in the current case study.

Patients presenting with subcutaneous nodules pose a challenge to practitioners, as this finding carries a very broad range of differential diagnoses [9]. The differential diagnosis of subcutaneous sarcoidosis includes rheumatoid nodules, xanthomas, cellulitis, subcutaneous granuloma annulare, foreign body granulomas, lipomas, cysts, or cutaneous manifestations of lymphoproliferative malignancies [10-13]. Also, erythema nodosum is one of the differentials of subcutaneous sarcoidosis, in our patient, we were able to distinguish these two skin findings histologically and clinically, as erythema nodosum usually presents symmetrically on the anterior surface of the tibia with severe pain [3]. On the other hand, subcutaneous sarcoidosis lesions of our patient were mainly found on the right upper limb with mild tenderness. In subcutaneous sarcoidosis patients, it is crucial to exclude other autoimmune conditions as they can either be a differential diagnosis of the lumps [13] or present as a co-existing condition. A study done by Ahmed et al. found that subcutaneous sarcoidosis can co-exist with other autoimmune conditions in 29% of cases, the autoimmune conditions found to be associated with subcutaneous sarcoidosis included Hashimoto thyroiditis, rheumatoid arthritis, ulcerative colitis, systemic lupus erythematosus, and sicca syndrome [8].

Acute phase reactants, such as ESR and CRP, have been used regularly in clinical practice in the workup of skin infections and even showed good results in predicting the severity of such conditions [14]. Also, ESR and CRP were found to be elevated in sarcoidosis patients [15]; similarly, this was noted in our patient. In the literature, case studies reported an overlap between subcutaneous sarcoidosis and skin infections. A case study of a 60-year-old male showed that subcutaneous sarcoidosis can present with findings that masqueraded as cellulitis; this patient presented with slightly erythematous, warm, and swollen right forearm and hand lesions that were increasing gradually in size over a period of two months without constitutional symptoms [10]. Another case report of a 54-year-old woman with a focal swelling in her thigh was misdiagnosed as cellulitis, Also, the ESR of this patient was noted to be slightly elevated [11]. Incorrect diagnosis of skin infections instead of sarcoidosis might lead to unnecessary prescription of antibiotics in such cases, which can lead to increased antibiotic resistance, duration of diseases, risk of complications, and healthcare costs [16]. The diagnosis of skin sarcoidosis based on signs and symptoms is considered quite challenging, as it can mimic a wide variety of illnesses. Hence, a skin biopsy should be done to reach a definitive diagnosis [17].

The treatment modality of choice in patients with subcutaneous sarcoidosis and systemic involvement is systemic steroids; other treatment options include hydroxychloroquine, methotrexate, clofazimine, intralesional glucocorticoids, thalidomide, dapsone, allopurinol, and minocycline [18]. Most patients treated with systemic steroids have complete remission, however, in some cases, hydroxychloroquine might be needed to treat cases that recur with the use of systemic steroids alone [12]. Alongside medical treatment, surgical excision was reported in other cases with complete resolution of the lesions [19]. In our case, both prednisone and hydroxychloroquine were used which led to remission of the nodules. The presence of subcutaneous sarcoidosis was found to be associated with a mild systemic disease and was linked to a favorable prognosis [12].

## Conclusions

In conclusion, subcutaneous nodules carry a wide range of differential diagnoses. This can delay the treatment of this condition. Subcutaneous sarcoidosis is one of the potential diseases that should be included in the differential diagnosis of presenting nodules.
